# Valp1, a Newly Identified Temperate Phage Facilitating Coexistence of Lysogenic and Non-Lysogenic Populations of *Vibrio anguillarum*

**DOI:** 10.3390/pathogens13040285

**Published:** 2024-03-27

**Authors:** Manuel Arce, Guillermo Venegas, Karla Paez, Simone Latz, Paola Navarrete, Mario Caruffo, Carmen Feijoo, Katherine García, Roberto Bastías

**Affiliations:** 1 Instituto de Biología, Pontificia Universidad Católica de Valparaíso, Valparaíso 2340000, Chile; manuelarce329@gmail.com (M.A.);; 2 Laboratory of Microbiology and Probiotics, Institute of Nutrition and Food Technology (INTA), University of Chile, Santiago 7830490, Chile; 3 Center for Research and Innovation in Aquaculture (CRIA), Universidad de Chile, Santiago 8820000, Chile; 4 Departamento de Ciencias Biológicas, Facultad de Ciencias de la Vida, Universidad Andres Bello, Santiago 8370146, Chile; 5 Instituto de Ciencias Biomédicas, Facultad de Ciencias de la Salud, Universidad Autónoma de Chile, Santiago 8910060, Chile

**Keywords:** bacteriophage, phage, vibriophage, *Vibro*, *Vibrio anguillarum*, telomeric phage

## Abstract

*Vibrio anguillarum* is a pathogen for several fish and shellfish species. Its ecology is influenced by diverse factors, including bacteriophages. Here, we identify and characterize a new temperate bacteriophage (Valp1) of *V. anguillarum*. Valp1 is a myovirus with a 60 nm head and a 90 nm contractile tail. Its double-stranded DNA genome of 42,988 bp contains 68 genes, including a protelomerase gene, typical of telomeric phages. Valp1 inhibits the growth of the virulent strain of *V. anguillarum* PF4, while the derived lysogenic strain P1.1 presents a slight reduction in its growth but is not affected by the presence of Valp1. Both strains present similar virulence in a larval zebrafish (*Danio rerio*) model, and only slight differences have been observed in their biochemical profile. Co-culture assays reveal that PF4 and P1.1 can coexist for 10 h in the presence of naturally induced Valp1, with the proportion of PF4 ranging between 28% and 1.6%. By the end of the assay, the phage reached a concentration of ~10^8^ PFU/mL, and all the non-lysogenic PF4 strains were resistant to Valp1. This equilibrium was maintained even after five successive subcultures, suggesting the existence of a coexistence mechanism between the lysogenic and non-lysogenic populations of *V. anguillarum* in conjunction with the phage Valp1.

## 1. Introduction

Members of the *Vibrionaceae* family thrive abundantly in marine and estuarine environments, including several pathogenic species responsible for infections in mollusks and humans [1,2]. Vibriosis is one of the most common infections for marine fish in their natural environment and aquaculture, with outbreaks steadily increasing in recent years [3,4]. Among species able to cause vibriosis, *V. anguillarum* is one of the most versatile marine pathogens, able to infect and cause mortalities in more than 50 fish species, some of which hold substantial economic significance [5]. The abundance of *V. anguillarum* in the environment is affected by several physicochemical factors, such as temperature and salinity [6], and biological factors, such as bacteriophages, their natural predators [7,8]. Bacteriophages can modulate the structure and abundance of bacterial communities through their lytic effect [9]. Temperate phages can also directly influence the evolution of their host through phenomena such as lysogenic conversion [10,11]. Therefore, it is relevant to study the interaction between temperate phages and their bacterial host, especially in bacteria that can be pathogenic, such as vibrios.

Within the genus *Vibrio*, several phages are known to increase the virulence of their hosts. A well-studied example is the toxigenic *Vibrio cholerae* and the filamentous temperate phage CTX, which encodes the toxin CTX [12]. Other examples include the phages VHML [13] and VHS1 [14], which infect the species *Vibrio harveyi* and are also recognized for their ability to enhance the virulence of their hosts through lysogeny. Nonetheless, the impact of temperate phages on their bacterial host is not limited to an increase in their virulence, and several other bacterial properties can be affected, influencing their physiology, lifestyle, and ecology [15,16]. Several prophages have been discovered and induced from different *V. anguillarum* strains, showing great genomic diversity and dynamics to transfer between different strains [17]. While other studies have shown that specific temperate phages are widely spread in the population of different *V. anguillarum* isolates [18]. Here, we show the characterization of the temperate phage vB_VaM_Valp1 (Valp1 thereafter) of *V. anguillarum* and its interactions with the bacterial host. This study contributes to understanding the role of phages in the biology of *V. anguillarum*.

## 2. Materials and Methods

### 2.1. Bacterial Strains and Cultivation

The bacterial strain *Vibrio anguillarum* PF4, widely studied [19,20,21], was used for phage isolation (wt strain). Strains PF8 and PF7 were obtained from our collection and were initially isolated with the PF4 strain [21]. The strains ATCC19264, LGM10939, NCIMB1875, NCIMB2129, NCIMB572, and NCIMB828 were obtained from international bacterial culture collections. The lysogenic strain P1.1 was obtained from a culture of PF4 infected with the Valp1 phage. All the bacterial strains used in this study were grown in AMS_LB medium (23.4 g/L NaCl, 24.7 g/L MgSO_4_ × 7H_2_O, 1.5 g/L KCl, and 1.43 g/L CaCl_2_ × 2H_2_O, 1% tryptone, 0.5% yeast extract, pH 6.5), solid medium was supplemented with 1.5% agar [7].

### 2.2. Phage Isolation and Propagation

Seawater samples from the Valparaíso Bay (Chile) were collected in sterile 1 L flasks for phage isolation. Samples were centrifuged (3000× *g*, 10 min.), filtered with 0.2 µm filters, and supplemented with 1% tryptone and 0.5% yeast extract. Then, for phage enrichment, the samples were inoculated with 500 µL of an exponential culture of the PF4 strain (O.D. 600 mn, ~0.5) and incubated for 24 h at 25 °C with shaking. After incubation, 500 µL was collected, centrifuged (3000× *g*, 10 min.), and filtered (0.2 µm) for phage detection using the standard double-layer agar assay [22] and the strain *V. anguillarum* PF4. An individual lytic plaque was isolated three consecutive times and then propagated in the strain PF4. For this purpose, an early exponential culture of the bacteria (O.D. 600nm, ~0.2) was infected with the Valp1 phage using an MOI of 10. After 24 h of incubation at 25 °C with agitation, the lysate was centrifuged and filtered through 0.2 µm filters. The lysate was stored at 4 °C and −80 °C with 30% glycerol. Phage concentrations were determined by the standard double-layer agar assay [22].

### 2.3. Phage Characterization

Infection curves were performed in multi-well plates infecting an early exponential culture of the corresponding bacteria with the Valp1 phage using an MOI of 10. The culture was incubated at 25 °C for 24 h, and bacterial growth was followed by measuring the O.D. 600 nm with a microplate spectrophotometer Infinite RM200 NanoQuant (TECAN, Männedorf, Switzerland). The same procedure was used to evaluate the growth of PF4 strains resistant to Valp1 infection but without the phage. The host range was determined with the spot test and the standard double-layer agar assay [23]. One-step growth curve was performed by infecting an early exponential culture of the bacteria with the phage at an MOI of 0.01. This mixture was incubated at 25 °C for 15 min and then centrifuged at 6000× *g* for 10 min. The supernatant with free phage was discarded while the pellet was resuspended in 20 mL of fresh liquid medium. Then, 20 µL was collected every 10 min for 70 min to determine the concentration of phage realised using the standard double-layer assay [24].

For the morphological and genomic characterization, the phage was first precipitated with 10% polyethylene glycol (PEG 8000) in 1 M NaCl at 4 °C for 10 h and then centrifuged at 10,000× *g* for 10 min, at 4 °C. The phage pellet was resuspended in 500 µL of S.M. buffer with chloroform 1%. This concentrated phage was used for transmission electron microscopy with negative stain using 2% aqueous uranyl acetate (pH 4.0) and a Phillips TECNAI 12, Biotwin. DNA extraction was performed by incubating the samples of concentrated phage with DNase (2 mg/mL) and RNase (100 mg/mL) for 1 h at 37 °C. Then, the samples were treated with proteinase K (500 mg/mL) for 15 min at 65 °C, and then sodium dodecyl sulfate (SDS) was added to a final concentration of 0.5% for 45 min of additional incubation at the same temperature. Then, the samples were treated twice with phenol/chloroform/isoamyl alcohol (25:24:1). The aqueous phase was recovered for DNA precipitation by adding 1/10 volume of 3 M sodium acetate and two volumes of frozen absolute ethanol. After centrifugation, the pellet was washed with ethanol 70% and resuspended in T.E. buffer [7].

The phage genome was sequenced with the Illumina Hiseq platform at Macrogen Inc. (Seoul, Republic of Korea). The sequences were assembled using the Galaxy CPT platform following the workflow phage genome assembler v2021.03 [25]. Genome annotation was performed using the Pharokka annotation tool [26], following a manual curation. The visualization of the phage genome was done with the online tool Proksee [27], and the phylogenetic analysis was performed with the online tool VipTree [28] and PhaBOX [29]. The GeneBank accession number for the Valp1 phage is OR500391.

### 2.4. Isolation and Characterization of Lysogenic Strain P1.1

Lysogenic strains were obtained from an infection curve of the PF4 strain with the Valp1 phage. Samples from the infection curve were inoculated in AMS_LB plates to isolate colonies resistant to Valp1 infection. The isolated colonies were cultivated at least three times in a solid medium without Valp1. The lysogenic nature of the P1.1 strain was determined by its phenotypical and molecular characteristics. First, infection curves to evaluate if the P1.1 strain (together with isolates P1.3, P1.4, P1.7, and P1.15) was immune to the infection by Valp1 were determined as explained before. Phages spontaneously released from a P1.1 culture were not able to form lytic plaques in the lysogenic strains but did form lytic plaques in a lawn of PF4 strains. The phage released by P1.1 was concentrated and observed by transmission electronic microscopy, as described before. The presence of Valp1 phage inside the strain P1.1 was also demonstrated with PCR using specific primers to amplify a 707 bp fragment of Valp1 endolysin gene (forward primer 5′-GGACGATCGGTAATGAAGGC-3′ and reverse primer 5′-CTGTATTGACCTGAGTGACGG-3′), and primers to amplify a 168 pb fragment of 16S RNA gene specific for vibrios (forward primer 5′-CCTGGTAGTCCACGCCGTAA-3′ and reverse primer 5′-CGAATTAAACCACATGCTCCA-3′) [30]. The PCR reaction was performed in a final volume of 12.5 µL with 4.2 µL of Sapphire Fast PCR Master mix (Takara Bio), 1.25 µL of each primer (10 µM), 1 µL of DNA, and 2.3 µL of nuclease-free water. The PCR program consisted of an initial denaturation step of 95 °C for 30 s, an annealing temperature of 55 °C for 30 s, and an elongation step at 72 °C for 2 min. The PCR products were visualized in a 1% agarose gel electrophoresis using SAFEVIEW plus (Fermelo, Santiago, Chile) and a 1 kb DNA ladder (New England BioLabs, Ipswich, MA, USA). For the RFLP analysis, the phage DNA was extracted as described before. The genomes were digested with the enzyme *Hinf*I (New England Biolabs) according to the manufacturer’s instructions. Fragments were separated by electrophoresis on a 8% polyacrylamide gel for 4 h at 70 V, and a 100 bp plus ladder (Bioneer, Daejeon, Republic of Korea) was used as molecular marker.

Biofilm production was evaluated in 96 multi-well plates, with 100 µL of 10-fold dilution of the corresponding bacterial culture at an O.D. 600 nm of 0.1. The plates were incubated for 24 h at 25 °C, and then the liquid from each well was discarded. The wells were washed with distilled water, and 125 µL of 1% crystal violet solution was added. The plate was incubated at room temperature for 15 min, and then the content of the plate was transferred to a new plate to measure the O.D. 550 nm in a plate reader Infinite RM200 NanoQuant (TECAN) [31]. Motility was evaluated in TSB plates supplemented with 0.4% agar and 2.3% NaCl. The plates were inoculated in the center with the corresponding bacterial culture (O.D. 600 nm, ~0.1). The motility halos were measured after 48 h of incubation at 25 °C. The metabolic profile of P1.1 strain was performed with the BIOLOG GEN III MICROPLATE test according to the manufacturer’s instructions [31].

Virulence assays were performed as described previously [19]. Briefly, TAB5 zebrafish (*Danio rerio*) larvae (n = 25) were transferred via a plastic Pasteur pipette into a well of a sterile 6-well tissue culture plate in a final volume of 12 mL of sterile E3 medium (1% NaCl; 0.17 mM KCl; 0.33 mM CaCl_2_; 0.33 Mm MgSO_4_; 0.05% methylene blue), with triplicates for each condition. Exponential bacterial cultures of *V. anguillarum* strains PF4 and P1.1 were pelleted and resuspended in a sterile E3 medium. For the larval challenge, 500 µL of the corresponding bacterial suspension was added to every well to reach a final concentration of 10⁷ CFU/mL (LD50). For control (nonchallenged larvae), only the E3 medium was inoculated. After 15 min of *V. anguillarum* exposition, all larvae were transferred to fresh E3 medium. Plates were incubated at 28 °C for 5 days. At day 3 post-infection (dpi), larvae were transferred into a new sterile 6-well tissue plate. To maintain good oxygenation, approximately 75% of the E3 medium was renewed daily. Mortality was evaluated daily in each group of zebrafish larvae, and dead larvae were removed. The experiments were independently repeated three times. The protocols with zebrafish larvae were approved by the local and central Committee for Ethics of Animal Experiments from INTA and the University of Chile (FCYT1-18-PN).

### 2.5. Co-Culture Experiments

Co-culture experiments between the strains PF4 and the lysogenic P1.1 were performed as follows. Overnight cultures of both strains grown in AMS_LB were diluted to ~10^7^ CFU/mL and used to inoculate with a 1:1 proportion of 100 mL of fresh liquid media. The co-culture was incubated at 25 °C with agitation for 10 h. Then, 1 mL of the co-culture samples was collected every h to determine the total bacteria concentration and phage concentration using the PF4 strain as a host. To estimate the proportion between the strains, PF4 and P1.1, 20 colonies were randomly selected from each time point. These bacteria were cultivated three consecutive times in AMS_LB plates to ensure purification from remaining phages. The identity of each colony was determined with PCR, as described before. Specific primers for Valp1 endolysin were used to identify the P1.1 strain, and specific primers for vibrios’ 16S RNA were used to identify strains P1.1 and PF4. Colonies identified as PF4 were evaluated for resistance to the Valp1 phage using the standard double-layer agar assay [22]. All experiments were performed in triplicate unless otherwise specified.

For subculture assays, after 10 h of the co-culture assay, 200 µL was collected to inoculate 10 mL of fresh liquid media. The subcultures were performed after two of the co-cultures replicates. The culture was incubated at 25 °C with agitation for 24 h, and the process was repeated for 5 days. At the end of each subculture, 1 mL was collected to determine the total bacteria concentration, and 20 colonies were randomly selected to estimate the proportion between PF4 and P1.1 by PCR as explained before. The proportions of PF4 and P1.1 presented in coexistence experiments correspond to the average proportion observed in each experimental replicate. The resistance to the Valp1 phage was evaluated in colonies identified as PF4, obtained from the different co-culture assays, as described above [22].

### 2.6. Analysis and Statistics

Survival rates of zebrafish larvae were analyzed with GraphPad Prism 6 software, using a nonparametric log-rank (Mantel–Cox) test to compare survival rates of different larval groups.

## 3. Results

### 3.1. Phage Characterization and Isolation

**Figure 1:**
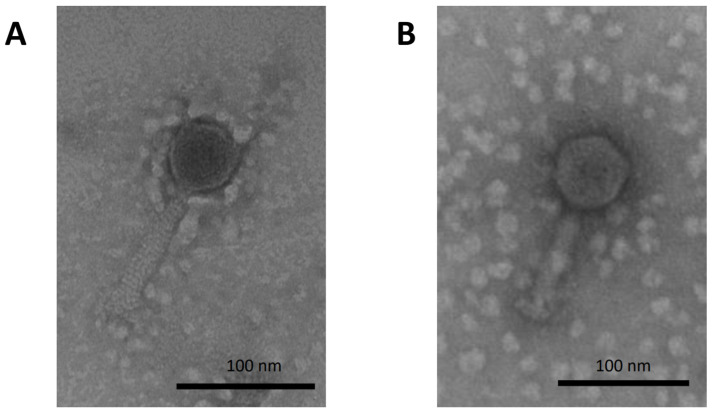
Transmission electron microscopy of Valp1 phage. (**A**) Morphology of Valp1 phage. (**B**) Morphology of Valp1 phage induced from *V. anguillarum* lysogenic strain P1.1.

Marine water samples from Valparaíso Bay were used to isolate phages against the *V. anguillarum* strain PF4 using enrichment and a double-layer agar assay. A new phage found in one of the samples was named Valp1 and characterized to evaluate its interaction with its bacterial host. Transmission electron microscopy showed that the Valp1 phage had a typical morphology of *Caudoviricetes* with an icosahedral head of approximately 60 nm in diameter and a contractile tail 90 nm long and 20 nm wide (Figure 1). The phage was able to infect only three out of nine *V. anguillarum* strains evaluated (Table S1), suggesting it has a narrow host range.

### 3.2. Genomic Characterization of Valp1 Phage

**Figure 2:**
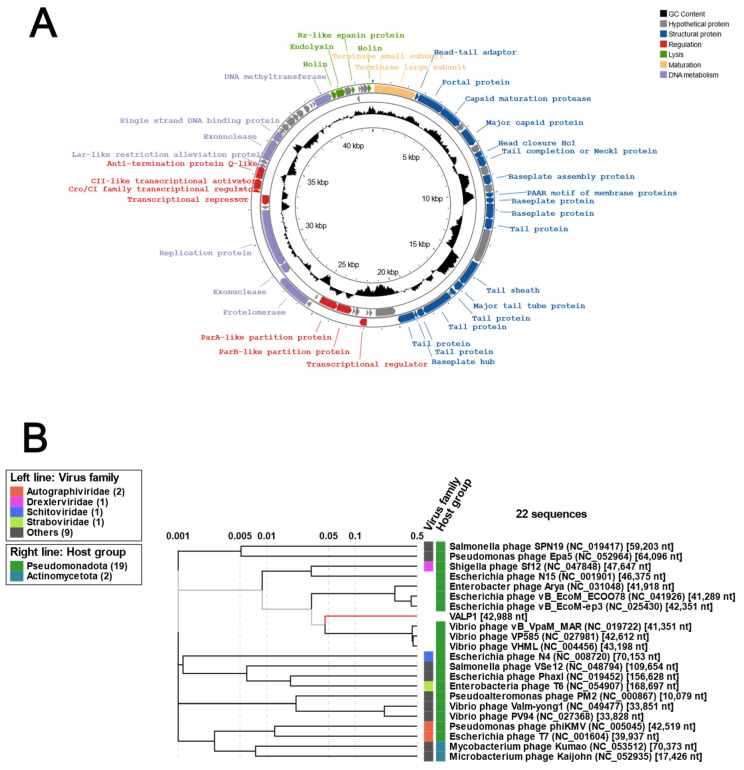
The genome of phage Valp1. (**A**) Genomic organization of Valp1 phage. The inner ring shows the genome size in kbp. The GC content can be observed in black. The genes with predicted annotations are colored and labeled according to the function or classification. Hypothetical proteins are not labeled. Concentric circles with different genes denote different reading frames. The genome map was created using the online tool Proksee [27]. (**B**) Proteomic tree of Valp1 with selected phages using the tool VipTree [28]. Valp1 is denoted with a red line. The predicted phage family and host group are shown together with the GenBank accession number for each phage. Complete phylogenetic analysis with 2043 viral sequences is presented in Figure S1.

As part of the characterization, the genome of Valp1 was fully sequenced and deposited in the NCBI database (accession number OR500391). The double-stranded DNA genome of Valp1 has a size of 42,988 bp and contains 68 putative genes (Figure 2A). The annotation details and putative functions of each coding sequence of Valp1 can be revised in Table S2. The analysis of Valp1 genome revealed that of the 68 potential genes, 38 were assigned with putative functions (55.8%) based on their amino acid sequence homology with proteins or conserved protein domains and motifs. Roughly 44% of the genes (30 genes) were categorized as hypothetical proteins, with 8.8% of them (6 genes) having no match in BLAST (Table S2). The Valp1 genome encodes genes related to functions, such as exonuclease recombination-associated protein, repressor proteins, exonucleases, ParA and ParB like proteins, and a protelomerase, often associated with telomeric bacteriophages. As illustrated in Figure 2A, the Valp1 genome harbors genes related to a lysogenic lifestyle cycle, such as a Cro/CI family transcriptional activator, CII-like transcriptional activator, and antitermination protein Q-like. Moreover, the phage also encodes other classical phage genes like structural proteins (head–tail adaptor, portal protein, capsid maturation protease, major capsid protein, head closure Hc1, tail completion of Neck1 protein, baseplate assembly protein, baseplate wedge subunit, baseplate protein, tail proteins, phage tail sheath, phage major tail tube protein, and minor tail proteins) and lysis (holin and endolysins), maturation (terminase, small and large subunits), and nucleic acid metabolism (DNA methyltransferase, single-strand DNA binding protein, Lar-like restriction alleviation protein, replication protein).

The phylogenetic analysis of Valp1 using the online tool VipTree, which creates a tree based on aminoacidic sequences, revealed that this phage was closely related to other *Vibrio* phages including the temperate vibriophage VHML (*Vibrio harveyi* Myovirus Like), the temperate and telomeric phage of *V. parahaemolyticus*, Vp58.5, and the myovirus of *V. parahaemolyticus*, vB_VparM_MAR (Figure 2B). Consistently, an analysis with the web tool PhaBOX showed that phage Valp1 belonged to the Casjensviridae family. All these analyses suggested that Valp1 could be a temperate telomeric phage of *V. anguillarum*.

### 3.3. Infection of V. anguillarum with Valp1 Phage

**Figure 3:**
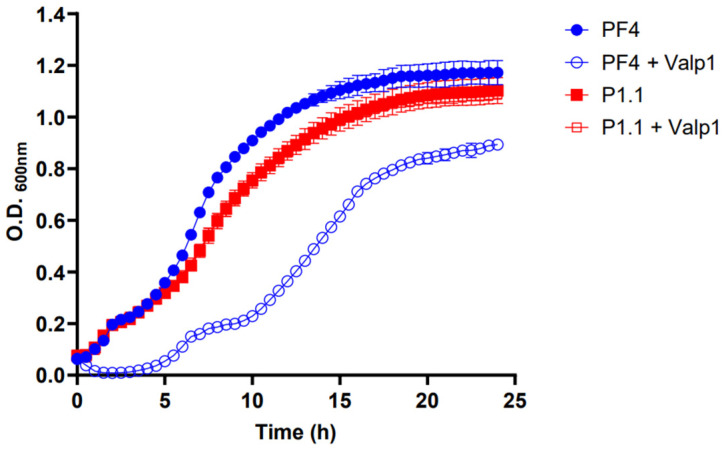
Infection curve of strains PF4 and P1.1 of *V. anguillarum* with the Valp1 phage. Infection curve of *V. anguillarum* strains PF4 and P1.1 in the presence of Valp1 phage (solid blue circle line: PF4 strain, empty blue circle line: PF4 strain + Valp1, solid red square line: P1.1 strain, empty red square line: P1.1 strain + Valp1). The bacteria were infected at the early exponential phase (O.D. 600 nm, ~0.1) with the Valp1 phage using an MOI of 10. Error bars indicate standard deviation.

To assess the impact of Valp1 on the growth of *V. anguillarum*, an infection curve was conducted. The results showed that the *V. anguillarum* strain PF4 exhibited delayed growth when infected with the phage compared to the uninfected bacteria. After 5 h of incubation, the infected bacteria resumed its growth; however, it was not able to reach the levels of the uninfected bacteria, even after 25 h of incubation (Figure 3). On the other hand, a one-step infection curve showed that Valp1 phage had a latency period of 20 minutes and a burst size of 234 PFU/cell (Figure S2). As the genome analysis indicated that Valp1 phage was a temperate phage, the resistant bacteria observed during the infection curve likely corresponded to lysogenic bacteria. To evaluate this point, five colonies were isolated from the phage infection curve, and the assay was repeated with these colonies. In the absence of the phage, the potential lysogenic bacteria exhibited a reduction in their growth compared to the *V. anguillarum* strain PF4 (Figure 3 and Figure S3). This reduction could be attributed to the energetic cost of supporting the replication of a telomeric prophage inside the cell, or to a spontaneous induction of the prophage. In contrast to the PF4 strain, the potential lysogenic strains did not experience any alteration in their growth in the presence of Valp1 (Figure 3 and Figure S3), suggesting that they were immune to phage infection. All these strains presented similar motility and capacity to produce a biofilm compared to the PF4 strain (Figure S4). The P1.1 strain was selected for a deeper characterization.

### 3.4. Characterization of the V. anguillarum Lysogenic Strain P1.1

The presence of Valp1 phage genome in the *V. anguillarum* strain P1.1 was confirmed by PCR using specific primers to amplify a fragment of the phage endolysin gene (Figure S5A). Additionally, to confirm that the phage induced from strain P1.1 corresponded to Valp1, an RFLP analysis was performed to compare the restriction patterns of the genomes of both phages. The results showed that indeed, both phages presented the same restriction pattern when digested with the *Hinf*I enzyme (Figure S5B). Although attempts to induce the P1.1 strain with UV or Mitomycin C to produce Valp1 were unsuccessful, spontaneous phage release was detected in the supernatant of the P1.1 strain using a bacterial lawn of PF4 strain. Observing the phage produced by P1.1 under electron microscopy revealed that it had the same morphology as the original Valp1 phage (Figure 1B). All these results reinforce the idea that *V. anguillarum* strain P1.1 is a lysogenic bacterium for the Valp1 phage.

The analysis of the metabolic capacities of the P1.1 strain using the BIOLOG system revealed that this strain did not exhibit significant alterations compared to the PF4 strain, except for 11 tests, corresponding to 11.7% of the total metabolic tests considered (Table S3). Some differences were associated with the ability to metabolize D-gluconic acid or gelatin, suggesting that the phage exerts some metabolic alterations in the lysogenic bacteria.

There are several reports about temperate phages affecting the virulence of their host bacteria. Therefore, we investigated whether the Valp1 phage had any effect on the virulence of *V. anguillarum*. To this end, we compared the virulence of PF4 and lysogenic P1.1 strains of *V. anguillarum* in a zebrafish model (*Danio rerio*). At 5 days postchallenge, larvae challenged with PF4 and P1.1 strains showed a lower survival rate (45.8% and 44.8%, respectively) compared to the unchallenged control larvae (85.3%) (*p* ≤ 0.001) (Figure S6). No statistically significant differences were observed in the survival rate of larvae challenged with PF4 and lysogenic P1.1 strains (*p* = 0.3790), suggesting that Valp1 does not induce significant changes in *V. anguillarum* virulence.

### 3.5. Coexistence between the V. anguillarum Strains PF4 and P1.1

**Figure 4:**
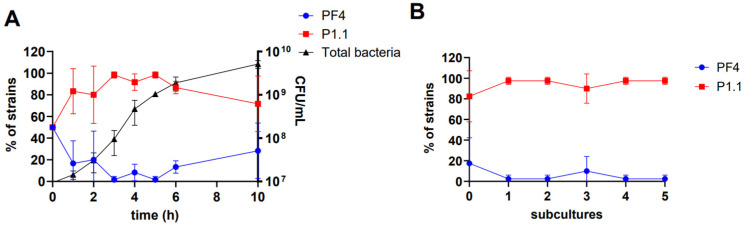
Coexistence dynamics between the *V. anguillarum* strains PF4 and P1.1. (**A**) Co-culture assay between the *V. anguillarum* strain PF4 (blue circle line) and the *V. anguillarum* lysogenic strain P1.1 (red square line). The black line with triangles represents the total bacteria. (**B**) Extended subcultures between the PF4 (blue circles) and P1.1 (red squares) strains. The proportions of PF4 and P1.1 presented correspond to the average proportion observed in each experimental replicate.

Considering that the lysogenic strain P1.1 spontaneously releases the Valp1 phage, we aimed to evaluate the outcome of a co-culture between the strains PF4 and P1.1. We expected the displacement of the PF4 strain from the co-culture, either through lysis or lysogenization by the Valp1 phage. However, during the assay, we observed that this bacterium could persist with the lysogenic strain P1.1 for up to 10 h in co-culture (Figure 4A). Within the first two h, the PF4 strain rapidly reduced its concentration to 20% of the total population, maintaining a proportion below 30% of the total bacterial population throughout the culture. In contrast, the P1.1 strain increased inversely in proportion, reaching a maximum of 98% of the total bacterial population after 3 h of culture. At the end of the trial, the total bacterial population reached 5 × 10^9^ CFU/mL, of which 71.6% corresponded to the lysogenic strain P1.1, while the phage reached a concentration of 2.6 × 10^8^ PFU/mL after 10 h (Figure S7).

To understand the persistence of the PF4 strain in the co-culture, we evaluated its resistance to the Valp1 phage. The results showed that the proportion of the PF4 strain resistant to the Valp1 phage increased progressively, reaching 100% after 4 h (Figure S7). This result explains the persistence of the PF4 strain in co-cultures with P1.1 despite the presence of the Valp1 phage in the medium.

Finally, we evaluated whether this coexistence phenomenon extended over time through a series of subcultures. For this purpose, samples were taken from the end of the co-culture trial and inoculated into a fresh culture medium. The results showed that PF4 could persist through different subcultures but in a small proportion within the total bacterial population that did not exceed 17% and, in some cases, only reached 2.5%. In contrast, the P1.1 strain completely dominated the bacterial population, with a proportion varying between 82.5% and 97.5% (Figure 4B). Similar to what was observed at the end of the co-culture experiment, the phage remained at a concentration ~10^7^ PFU/mL, and all the PF4 colonies recovered were resistant to the Valp1 phage.

## 4. Discussion

Bacteriophages exert a significant influence on the ecology and evolution of marine bacteria with substantial repercussions on their fitness and virulence [9,11,15]. This influence is particularly important for pathogenic bacteria, such as *V. anguillarum*, which is known to cause damage in the aquaculture industry [5]. In this study, we identified and characterized the Valp1 phage, a temperate phage of *V. anguillarum*, which belongs to the Casjensviridae family and possesses morphological characteristics of the myovirus phages.

It has been described that temperate bacteriophages can confer greater virulence to bacteria of the genus Vibrio [12,13,14]. This phenomenon was also reported for the temperate phage VHML, which infects V. harveyi and is related to the Valp1 phage [13]. However, our results show that the Valp1 phage does not increase the virulence of V. anguillarum in a zebrafish model, nor were any genes found in its genome that could be associated with virulence. Although this animal model has been previously used to evaluate the virulence of V. anguillarum strains [19,32], we cannot rule out the possibility that other fish, such as sea bass (Dicentrarchus labrax) larvae, may be more sensitive to differentiate the virulence of the two V. anguillarum strains [7,33].

On the other hand, it has been observed that temperate phages can affect their host bacteria in multiple ways that are not exclusively related to virulence [15,16]. In this sense, our results show that strains lysogenized with Valp1 do not alter motility or biofilm formation. However, they exhibit some modifications in their metabolic profile, which was also previously observed with the VHML phage [34]. It has been speculated that the presence of a gene that encodes a DNA methyltransferase in this type of phage could contribute to the phenotypic changes observed in lysogenic bacteria [34,35,36]. Phage Valp1 also has a gene that encodes a DNA methyltransferase that has a 61% and 58% amino acid identity with genes present in phages VHML and VP58.5, respectively. It is possible that this gene plays a role in the few phenotypic changes observed in P1.1. It is possible that Valp1 affects its bacterial host in other ways, but fully characterizing this interaction would require countless assays.

The analysis of the Valp1 genome revealed the presence of a protelomerase and genes that encode the parA/parB partitioning system. These types of genes are characteristic of telomeric phages such as VP58.5, VHML, and N-15 [35,36,37], which are also phylogenetically close to Valp1. This suggests that Valp1 could also be a telomeric phage; however, to confirm this, it would be necessary to experimentally verify that it persists with the bacteria in the form of a linear plasmid, as has been reported in other cases [35,38]. Persisting as a linear plasmid inside the bacteria could mean an advantage for this type of phage since they would not be subjected to the restrictions that have prophages integrated into bacterial chromosomes. Furthermore, plasmidial prophages could be transferred horizontally by conjugation, as has been observed in other types of plasmidial phages [39].

A noteworthy aspect of our results is the persistence of the PF4 strain in co-culture with the lysogenic strain P1.1 in the presence of the Valp1 phage. This phenomenon has been explored by other authors in *E. coli* and the lambda phage, where it was observed that under similar conditions, a predominance of lysogenic bacteria and non-lysogenic strains resistant to the phage also occurred [40]. However, in that case, both bacterial population maintained a similar proportion. As reported, the dynamics and equilibrium in such microbial communities depend on various factors, such as the induction rate of the lysogenic strain or the growth rates of the different bacterial populations present in the culture [41]. In this case, Valp1-resistant PF4 strains obtained from these assays did not show significant differences in their growth compared to the parental PF4 strain (Figure S8). Future studies will help elucidate how these or other parameters contribute to the coexistence between these bacterial populations.

## 5. Conclusions

This study introduces the discovery and detailed examination of Valp1, a newly identified temperate bacteriophage infecting *V. anguillarum*. Interestingly, while Valp1 does not modify the pathogenicity of its host in a zebrafish model, it does induce alterations in the metabolic functions of lysogenized bacteria. Furthermore, we observed that non-lysogenic bacterial populations are capable of coexisting with lysogenic ones, likely due to a developed resistance to Valp1. This suggests a complex interplay and coexistence mechanism among bacterial populations facilitated by the presence of Valp1, highlighting the intricate dynamics of phage-host interactions.

## Data Availability

All the data generated are available at the following link 10.6084/m9.figshare.25215236. The Valp1 genome is available in NCBI GeneBank under the accession number OR500391.

## Supplementary Materials

The following supporting information can be downloaded at: https://www.mdpi.com/article/10.3390/pathogens13040285/s1, Figure S1: Proteomic tree of Valp1 with 2043 viral sequences using the tool VipTree. The position of Valp1 is denoted by a red star and a red line in the tree. Viral families are denoted with colored boxes in the middle circle according to the inset. The taxonomy groups at the phylum level (except for Proteobacteria at the class level)for each bacterial host of the corresponding virus are denoted with colored boxes in the external circle according to the inset; Table S1. Host range of phage Valp1 in nine different V. anguillarum strains; Figure S2: A one-step growth curve with the V. anguillarum strain PF4 and the Valp1 phage. According to the results, the phage has a latency time of 20 min and a burst size of 234 PFU/mL. Error bars indicate standard deviation from triplicates; Table S2: Annotation of Valp1 genome. The genome is deposited in GeneBank under the accession number OR500391; Figure S3: Infection curve of V. anguillarum strain PF4 (Square) and the lysogenic strains P1.1 (triangle), P1.3 (circle), P1.4 (inverted triangle), P1.7 (diamond), P1.15 (asterisk) in the presence (solid) or absence (empty) of Valp1 phage. The curves for the strains PF4 and P1.1 are remarked with blue and red, respectively. The bacteria were infected at the early exponential phase (D.O 600 nm ~0.1) with the Valp1 phage using a MOI of 10. Error bars indicate standard deviation; Table S3: Differences in biochemical profile of PF4 strain and lysogenic strain P1.1 of V. anguillarum with BIOLOG GEN III; Figure S4: Phenotypic characterization of lysogenic strains of V. anguillarum compared to PF4. (A) Biofilm formation of different lysogenic strains of V. anguillarum for the phage Valp1. The assay was performed in quadruplicates in 96 multi-well plates, staining the exopolysaccharide of the biofilm with crystal violet as described in the material and methods sections. Error bars represent standard deviations. (B) Motility halo diameter for the different lysogenic strains of V. anguillarum for the phage Valp1. The test was conducted in a TSB medium supplemented with 0.4% agar and 2.3% NaCl. Cultures were incubated for 48 h at 25 °C. Error bars represent the standard deviation; Figure S5: Detection of Valp1 genome inside V. anguillarum lysogenic strain P1.1. (A) The presence of the phage genome was detected with PCR and specific primers to amplify a fragment of the endolysin gene (707 bp). Primers to amplify a fragment of the Vibrio 16S RNA gene (168 bp) were used as a control. Fragments were visualized in 1 % agarose gel electrophoresis using SAFEVIEW plus (Fermelo) and 100 bp DNA ladder (New England BioLabs). (B) To confirm that phage Valp1 corresponds to the same phage that is spontaneously induced from P1.1, an RFLP was performed with the genomes of both phages (phage Valp1 and phage induced from P1.1) using the enzyme *Hinf*I (New England Biolabs). Fragments were separated by electrophoresis on an 8% polyacrylamide gel for 3 h at 70 V using a 100 bp plus ladder (Bioneer); Figure S6: Effect of VALP1 on the virulence of V. anguillarum. Survival rate of zebrafish (Danio rerio) larvae after being challenged with wild type PF4 and lysogenic 1.1 V. anguillarum strains (the figure shows the average of in three independent experiments). The blue line with circles: uninfected unchallenged larvae (control group), the red lines with squares: larvae challenged by immersion with 107 CFU/mL of V. anguillarum PF4, the green lines with triangles: larvae challenged by immersion with 107 CFU/mL of V. anguillarum P1.1. dpc: days post-challenge. Survival of challenged larvae was lower compared to non-challenged larvae (*p* ≤ 0.001). No differences were observed in the survival rate of larvae challenged with wild-type PF4 and lysogenic P1.1 strains (*p* = 0.3790).; Figure S7: Proportion of V. anguillarum strain PF4 resistant to phage Valp1 and induction of Valp1 phage during co-culture experiments. The proportion of PF4 strains resistant to the infection of Valp1 (black triangles) was evaluated during the co-culture with P1.1 strain through the standard double agar layer method. Similarly, the concentration of phage Valp1 (Empty squares) produced by induction during the co-culture experiments was evaluated through the standard double agar layer method using the PF4 strain as indicator bacteria. The error bars indicate the standard deviation and Figure S8: Growth curve of parental strain PF4 and different Valp1-resistant PF4 strains. The growth of the bacteria was followed by measuring the O.D. 600 nm in a multi-well plate reader. The error bars indicate the standard deviation.
