# Supplementary material for: Valp1, a Newly Identified Temperate Phage Facilitating Coexistence of Lysogenic and Non-Lysogenic Populations of Vibrio anguillarum
Source: Pathogens. 2024 Mar 27;13(4):285. doi: 10.3390/pathogens13040285 (PMC11054321; doi:10.3390/pathogens13040285)
Supplement: Supplementary file 1 [file pathogens-13-00285-s001.zip › Supplementary figures Valp1 R1.pdf]

Valp1, a Newly Identified Temperate Phage Facilitating Coexistence of Lysogenic and Non-Lysogenic Populations of *Vibrio anguillarum*

Manuel Arce [1], Guillermo Venegas [1], Karla Paez [1], Simone Latz [1], Paola Navarrete [2], Mario Caruffo [2,3], Carmen Feijoo [4], Katherine García [5] and Roberto Bastías [1,*]

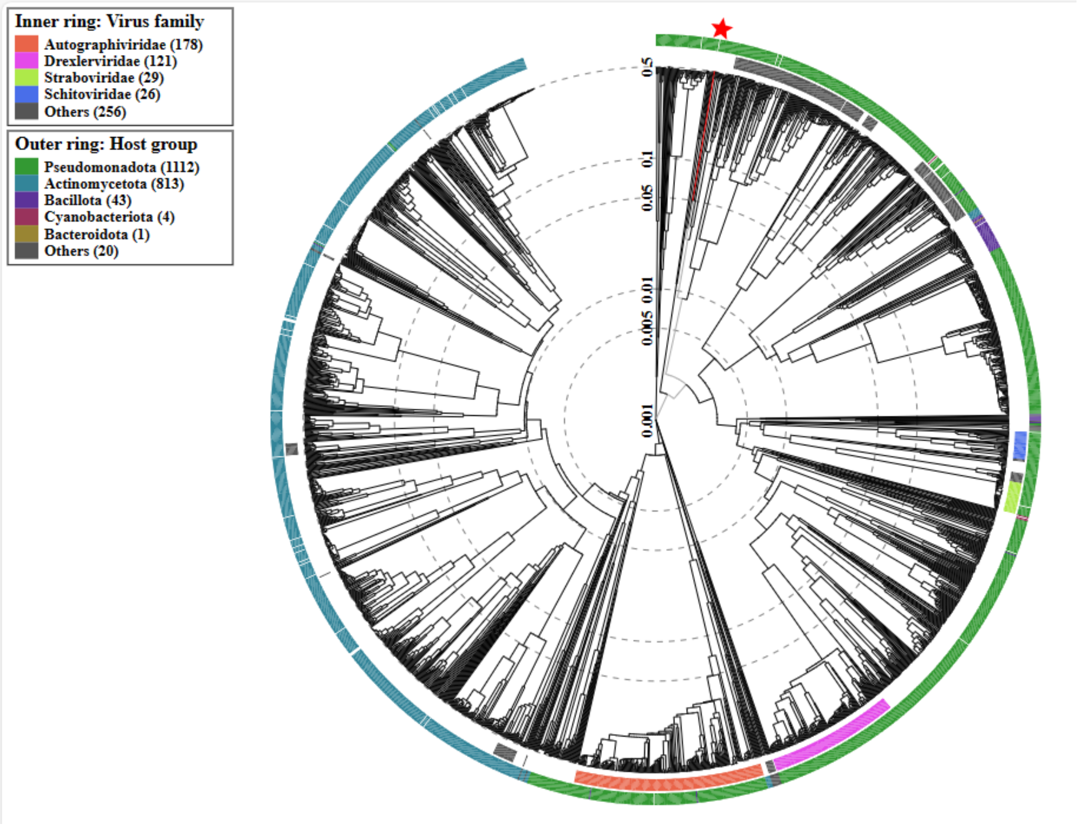

Figure S1. Proteomic tree of Valp1 with 2043 viral sequences using the tool VipTree. The position of Valp1 is denoted by a red star and a red line in the tree. Viral families are denoted with colored boxes in the middle circle according to the inset. The taxonomy groups at the phylum level (except for Proteobacteria at the class level)for each bacterial host of the corresponding virus are denoted with colored boxes in the external circle according to the inset.

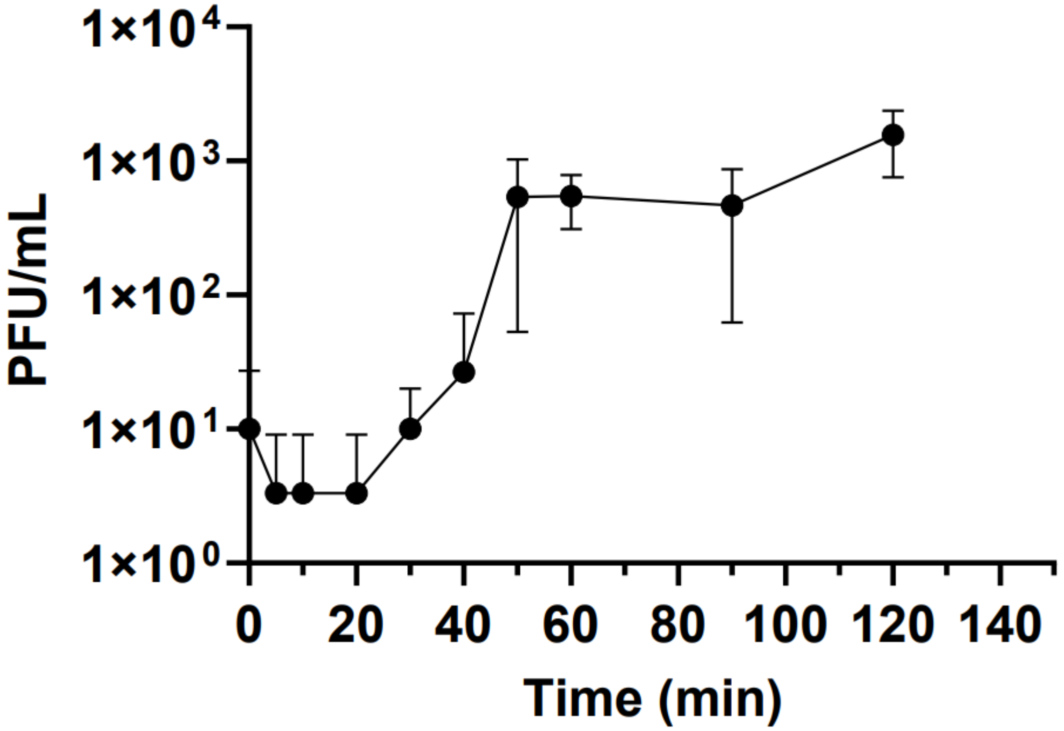

Figure S2. A one-step growth curve with the *V. anguillarum* strain PF4 and the Valp1 phage. According to the results, the phage has a latency time of 20 min and a burst size of 234 PFU/mL. Error bars indicate standard deviation from triplicates.

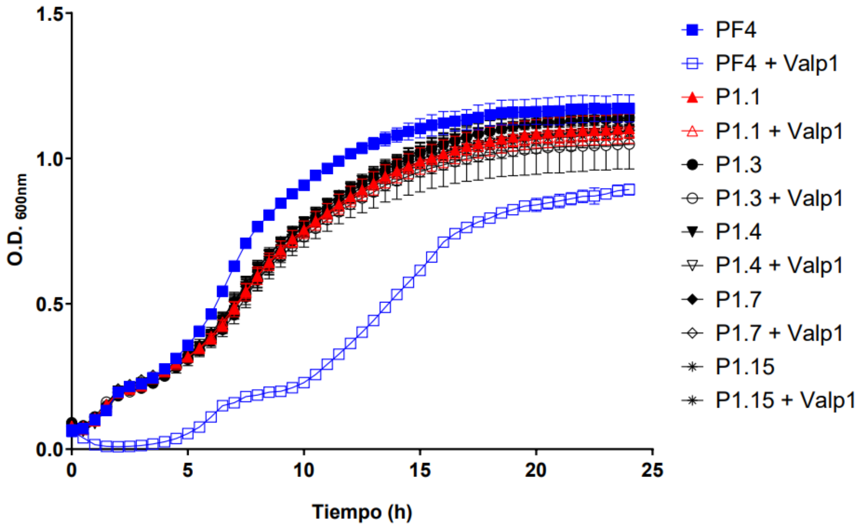

Figure S3. Infection curve of *V. anguillarum* strain PF4 (Square) and the lysogenic strains P1.1 (triangle), P1.3 (circle), P1.4 (inverted triangle), P1.7 (diamond), P1.15 (asterisk) in the presence (solid) or absence (empty) of Valp1 phage. The curves for the strains PF4 and P1.1 are remarked with blue and red, respectively. The bacteria were infected at the early exponential phase (D.O 600 nm ~0.1) with the Valp1 phage using a MOI of 10. Error bars indicate standard deviation.

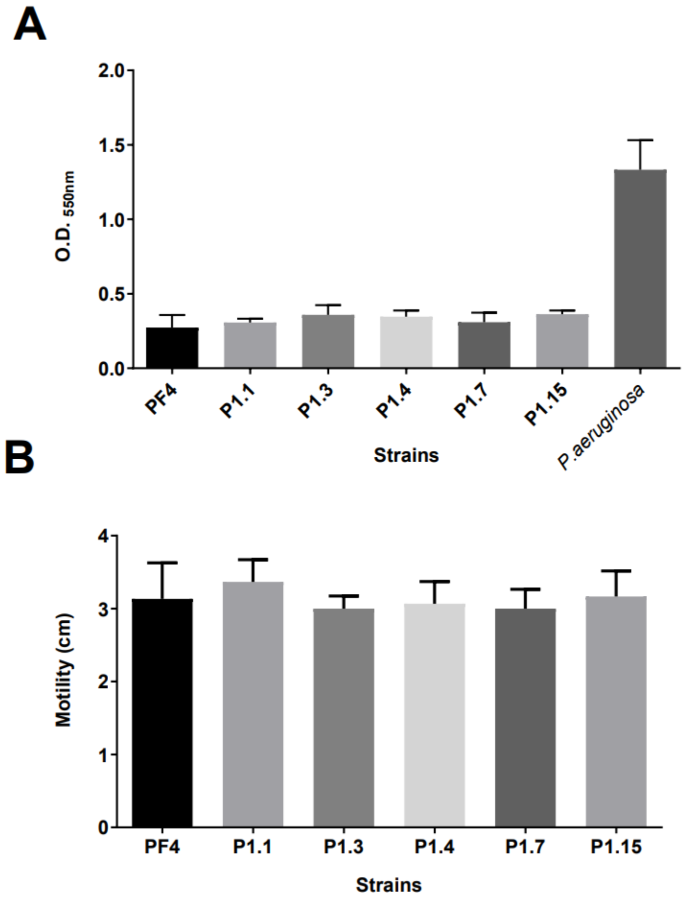

Figure S4. Phenotypic characterization of lysogenic strains of *V. anguillarum* compared to PF4. A) Biofilm formation of different lysogenic strains of *V. anguillarum* for the phage Valp1. The assay was performed in quadruplicates in 96 multi-well plates, staining the exopolysaccharide of the biofilm with crystal violet as described in the material and methods sections. Error bars represent standard deviations. B) Motility halo diameter for the different lysogenic strains of *V. anguillarum* for the phage Valp1. The test was conducted in a TSB medium supplemented with 0.4% agar and 2.3% NaCl. Cultures were incubated for 48 h at 25 °C. Error bars represent the standard deviation.

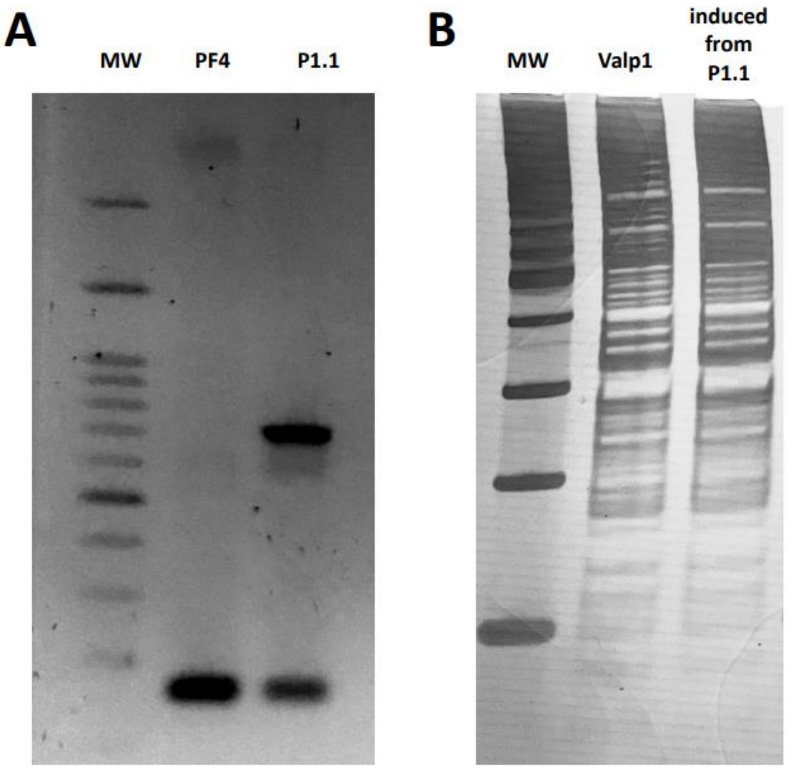

Figure S5. Detection of Valp1 genome inside *V. anguillarum* lysogenic strain P1.1. **A)** The presence of the phage genome was detected with PCR and specific primers to amplify a fragment of the endolysin gene (707 bp). Primers to amplify a fragment of the *Vibrio* 16S RNA gene (168 bp) were used as a control. Fragments were visualized in 1 % agarose gel electrophoresis using SAFEVIEW plus (Fermelo) and 100 bp DNA ladder (New England BioLabs). **B)** To confirm that phage Valp1 corresponds to the same phage that is spontaneously induced from P1.1, an RFLP was performed with the genomes of both phages (phage Valp1 and phage induced from P1.1) using the enzyme *Hinf* I (New England Biolabs). Fragments were separated by electrophoresis on an 8% polyacrylamide gel for 3 hours at 70 V using a 100 bp plus ladder (Bioneer).

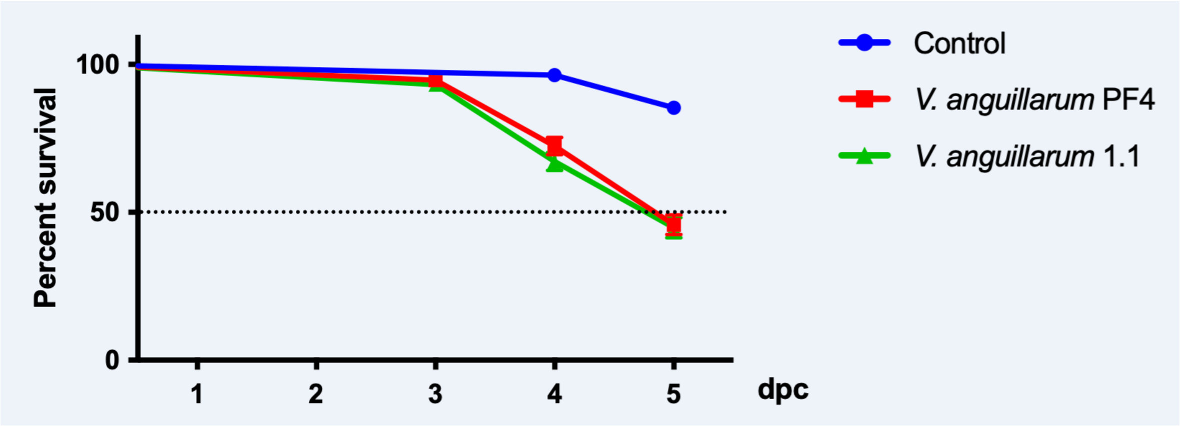

Figure S6. Effect of VALP1 on the virulence of *V. anguillarum*. Survival rate of zebrafish (*Danio rerio*) larvae after being challenged with wild type PF4 and lysogenic 1.1 *V. anguillarum* strains (the figure shows the average of in three independent experiments). The blue line with circles: uninfected unchallenged larvae (control group), the red lines with squares: larvae challenged by immersion with 107 CFU/mL of *V. anguillarum* PF4, the green lines with triangles: larvae challenged by immersion with 107 CFU/mL of *V. anguillarum* P1.1. dpc: days post-challenge. Survival of challenged larvae was lower compared to non-challenged larvae ($P \leq 0.001$). No differences were observed in the survival rate of larvae challenged with wild-type PF4 and lysogenic P1.1 strains ($P = 0.3790$).

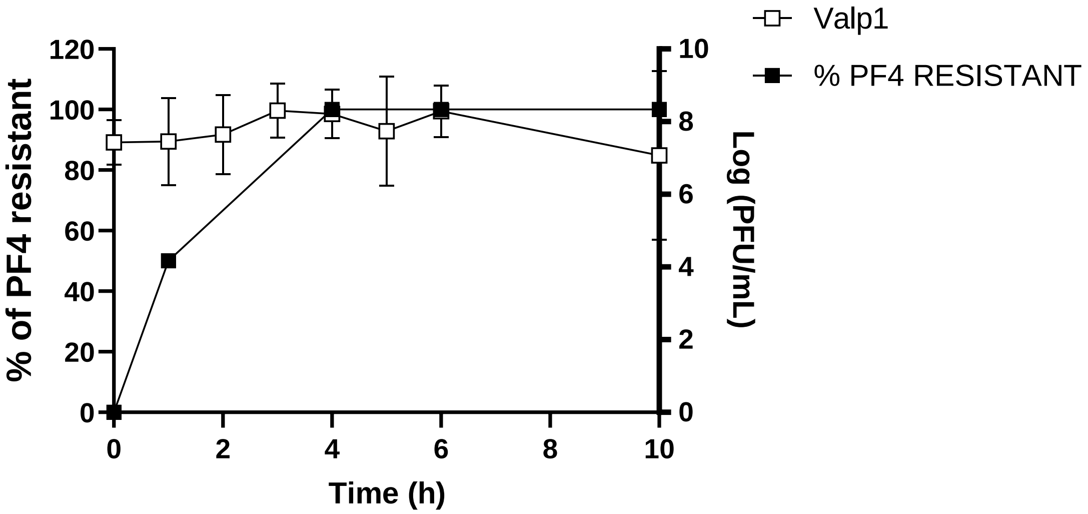

Figure S7. Proportion of *V. anguillarum* strain PF4 resistant to phage Valp1 and induction of Valp1 phage during co-culture experiments. The proportion of PF4 strains resistant to the infection of Valp1 (black triangles) was evaluated during the co-culture with P1.1 strain through the standard double agar layer method. Similarly, the concentration of phage Valp1 (Empty squares) produced by induction during the co-culture experiments was evaluated through the standard double agar layer method using the PF4 strain as indicator bacteria. The error bars indicate the standard deviation.

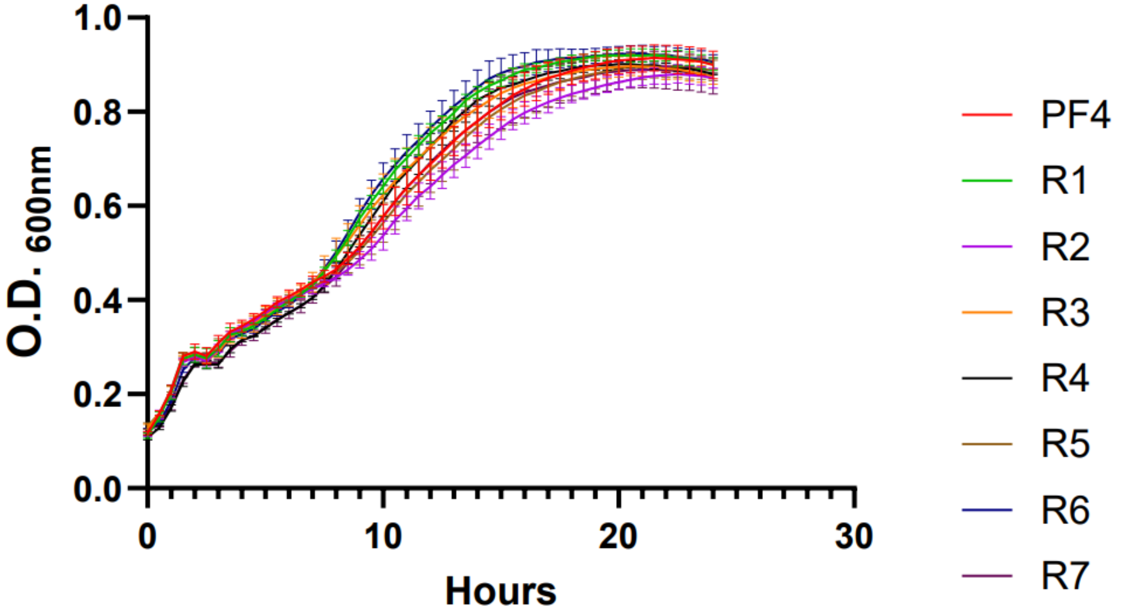

Figure S8. Growth curve of parental strain PF4 and different Valp1-resistant PF4 strains. The growth of the bacteria was followed by measuring the O.D. 600 nm in a multi-well plate reader. The error bars indicate the standard deviation.
