# Supplementary material for: Valp1, a Newly Identified Temperate Phage Facilitating Coexistence of Lysogenic and Non-Lysogenic Populations of Vibrio anguillarum
Source: Pathogens. 2024 Mar 27;13(4):285. doi: 10.3390/pathogens13040285 (PMC11054321; doi:10.3390/pathogens13040285)
Supplement: Supplementary file 1 [file pathogens-13-00285-s001.zip › Supplementary Tables.pdf]

Valp1, a Newly Identified Temperate Phage Facilitating Coexistence of Lysogenic and Non-Lysogenic Populations of *Vibrio anguillarum*

Manuel Arce [1], Guillermo Venegas [1], Karla Paez [1], Simone Latz [1], Paola Navarrete [2], Mario Caruffo [2,3], Carmen Feijoo [4], Katherine García [5] and Roberto Bastías [1,*]

Table S1. Host range of phage Valp1 in nine different *V. anguillarum* strains.

	***V. anguillarum* strain**								
	PF4	PF8	PF7	ATCC 19264	LGM 10939	NCIMB 1875	NCIMB 2129	NCIMB 572	NCIMB 828
**Lysis[a]**	+	+	-	-	-	-	-	+	-

[a] (+) indicates bacterial lysis; (-) indicates no lysis.

Table S2. Annotation of Valp1 genome. The genome is deposited in GeneBank under the accession number OR500391.

ORF	Nucleotide position	Putative function	BLAST hit [Accession number]	% Aminoacidic identity	E-value
CDS_01	90-296	Holin	Hypothetical protein *Vibrio* [WP_016786046.1]	100	3,00E-39
CDS_02	283-555	Hypothetical protein	Hypothetical protein *Vibrio* [WP_016786045.1]	100	1,00E-61
CDS_03	527-706	Hypothetical protein	Hypothetical protein *Vibrio crassostreae* [WP_132941303.1]	100	5,00E-34
CDS_04	709-831	Hypothetical protein	Hypothetical protein *Vibrio crassostreae* WP_282597755.1	100	2,00E-21
CDS_05	852-1010	Hypothetical protein	No match	-	-
CDS_06	1048-1227	Rz-like spanin	Hypothetical protein *Vibrio crassostreae* [TCN02923.1]	100	1,00E-34
CDS_07	1229-1633	Hypothetical protein	DUF2570 family protein *Vibrio crassostreae* [WP_132941302.1]	100	1,00E-93
CDS_08	1635-2159	Endolysin	Lysozyme *Vibrio* [WP_016786041.1]	100	3,00E-127
CDS_09	2159-2437	Holin	HP1 family phage holin *Vibrio* [WP_016786040.1]	100	4,00E-57
CDS_10	2461-3507	DNA methyltransferase	DNA methyltransferase *Vibrio* [WP_016786039.1]	100	0.0
CDS_11	3522-3647	Hypothetical protein	DNA-directed RNA polymerase subunit P *Vibrio* phage vB_Va_Val-yong3 [UVD31987.1]	92	1,00E-15
CDS_12	3715-3807	Hypothetical protein	No match	-	-
CDS_13	3923-4219	Hypothetical protein	Hypothetical protein *Vibrio crassostreae* [WP_132941301.1]	98	1,00E-64
CDS_14	4362-4724	Hypothetical protein	Hypothetical protein *Vibrio crassostreae* [WP_132941300.1]	100	1,00E-81

CDS_15	4748-5014	Hypothetical protein	Hypothetical protein EDB35_13114 *Vibrio crassostreae* [TCN02916.1]	100	5e-58
CDS_16	5014-5508	Hypothetical protein	DUF4406 domain-containing protein *Vibrio crassostreae* [WP_207904214.1]	99	2,00E-117
CDS_17	5535-5891	Hypothetical protein	Hypothetical protein *Vibrio* [WP_132941298.1]	100	3,00E-82
CDS_18	5881-6051	Hypothetical protein	Hypothetical protein *Vibrio crassostreae* [WP_165922799.1]	100	4,00E-33
CDS_19	6054-6149	Hypothetical protein	No match	-	-
CDS_20	6140-6727	Single strand DNA binding protein	Single-stranded DNA-binding protein *Vibrio crassostreae* [WP_132941297.1]	100	1e-139
CDS_21	6727-7992	Exonuclease recombination-associated protein	Recombination-associated protein RdgC *Vibrio crassostreae* [WP_132941296.1]	99	0.0
CDS_22	8006-8215	Hypothetical protein	Hypothetical protein *Vibrio crassostreae* [WP_132941295.1]	100	2,00E-43
CDS_23	8230-8445	Lar-like restriction alleviation protein	Hypothetical protein CGH10_22345 *Vibrio parahaemolyticus* [TOP74123.1]	91	1,00E-41
CDS_24	8429-9157	Anti-termination protein Q-like	Bacteriophage antitermination protein Q *Vibrio crassostreae* [WP_132941294.1]	99	1,00E-178
CDS_25	9169-9783	CII-like transcriptional activator	Hypothetical protein *Vibrio crassostreae* [WP_132941293.1]	100	1,00E-150
CDS_26	9780-9977	Cro/CI family transcriptional regulator	Cro/CI family transcriptional regulator *Vibrio crassostreae* [WP_132941292.1]	100	1,00E-38
CDS_27	10094-10717	Transcriptional repressor	Phage repressor protein C with HTH and peptisase S24 domain *Vibrio crassostreae* [TCN02906.1]	99	2,00E-147

CDS_28	10805-10987	Hypothetical protein	Hypothetical protein CGH10_24535 *Vibrio parahaemolyticus* [TOP71479.1]	73	5,00E-21
CDS_29	11050-14712	Replication protein	PriCT-2 domain-containing protein *Vibrio crassostreae* [WP_132941290.1]	99	0.0
CDS_30	14713-15327	Exonuclease	3'-5' exoribonuclease *Vibrio crassostreae* [WP_132941289.1]	100	1,00E-150
CDS_31	15362-17404	Protelomerase	Protelomerase family protein *Vibrio crassostreae* [WP_243701244.1]	99	0.0
CDS_32	17461-17553	Hypothetical protein	No match	-	-
CDS_33	17555-17653	Hypothetical protein	No match	-	-
CDS_34	17662-17802	Hypothetical protein	hypothetical protein *Vibrio sp*. 1F_97 [AKN39688.1]	86	2,00E-17
CDS_35	18025-19191	ParA-like partition protein	AAA family ATPase *Vibrio cortegadensis* [WP_261890242.1]	99	0.0
CDS_36	19188-20141	ParB-like partition protein	ParB/RepB/Spo0J family partition protein *Vibrio cyclitrophicus* [WP_016786072.1]	98	0.0
CDS_37	20212-20367	Hypothetical protein	Hypothetical protein *Vibrio* [WP_165922801.1]	100	2,00E-29
CDS_38	20394-20648	Hypothetical protein	Hypothetical protein *Vibrio crassostreae* [WP_132941325.1]	96	3,00E-52
CDS_39	20699-21136	Transcriptional regulator	Helix-turn-helix transcriptional regulator *Vibrio* [WP_016786069.1]	99	2,00E-72
CDS_40	21106-21231	Hypothetical protein	No match	-	-
CDS_41	21281-21463	Hypothetical protein	Hypothetical protein *Vibrio* [WP_016786068.1]	98	1,00E-33
CDS_42	21673-22956	Hypothetical protein	SGNH/GDSL hydrolase family protein *Shewanella oneidensis* [WP_011072650.1]	50	8,00E-132

CDS_43	22953-23957	Tail protein	Contractile injection system protein, VgrG/Pvc8 family *Vibrio crassostreae* [WP_132941322.1]	99	0.0
CDS_44	23948-24121	Baseplate hub	Tail protein X *Vibrio* [WP_016785912.1]	100	2e-33
CDS_45	24129-24533	Tail protein	Phage tail protein *Vibrio* [WP_132941321.1]	100	5,00E-94
CDS_46	24533-26323	Tail protein	Phage tail protein *Vibrio crassostreae* [WP_132941320.1]	99	0.0
CDS_47	26304-26432	Hypothetical protein	Hypothetical protein EDB35_13146 *Vibrio crassostreae* [TCN02948.1]	97	5,00E-20
CDS_48	26429-26719	Tail protein	Phage tail assembly protein *Vibrio crassostreae* [WP_132941319.1]	98	6,00E-62
CDS_49	26789-27304	Major tail tube protein	Phage major tail tube protein *Vibrio* [WP_132700124.1]	100	4,00E-119
CDS_50	27304-28770	Tail sheath protein	Phage tail sheath subtilisin-like domain-containing protein *Vibrio alginolyticus* [WP_258510186.1]	98	0.0
CDS_51	28831-30816	Hypothetical protein	SGNH/GDSL hydrolase family protein *Shewanella oneidensis* [WP_011072650.1]	60	0.0
CDS_52	30822-31454	Tail protein	TPA: phage tail protein I *Vibrio parahaemolyticus* [HCG8352157.1]	76	9,00E-118
CDS_53	31451-32344	Baseplate protein	Baseplate J/gp47 family protein *Vibrio chagasii* [WP_150897831.1]	91	0.0
CDS_54	32344-32682	Baseplate protein	GPW/gp25 family protein *Vibrio navarrensis* [WP_193246663.1]	93	2,00E-70
CDS_55	32688-32978	PAAR motif of membran proteins	PAAR domain-containing protein *Vibrio cyclitrophicus* [WP_016785895.1]	96	2,00E-57

CDS_56	32966-33448	Hypothetical protein	Hypothetical protein *Paraglaciecola sp.* [WP_299075741.1]	95	8,00E-108
CDS_57	33448-34023	Baseplate assembly protein	Phage baseplate assembly protein V *Vibrio alginolyticus* [WP_258510196.1]	97	4,00E-134
CDS_58	34016-34546	Hypothetical protein	Hypothetical protein *Vibrio* [WP_016785892.1]	100	6,00E-128
CDS_59	34543-35133	Tail completion or Neck1 protein	Hypothetical protein *Vibrio crassostreae* [WP_132941331.1]	100	3,00E-139
CDS_60	35096-35458	Head closure Hc1	Head-tail joining protein *Vibrio crassostreae* [WP_132941310.1]	99	7,00E-80
CDS_61	35448-35876	Hypothetical protein	Hypothetical protein *Vibrio crassostreae* [WP_132941309.1]	99	5,00E-96
CDS_62	35932-36963	Major capsid protein	Major capsid protein *Vibrio crassostreae* [WP_132941308.1]	100	0.0
CDS_63	37026-37364	Hypothetical protein	Hypothetical protein *Vibrio* [WP_016786052.1]	99	5,00E-72
CDS_64	37364-38674	Capsid maturation protease	S49 family peptidase *Vibrio cyclitrophicus* [WP_016786051.1]	99	0.0
CDS_65	38637-40241	Portal protein	Phage portal protein *Vibrio* [WP_132941305.1]	99	0.0
CDS_66	40241-40441	Head-tail adaptor Ad1	gpW family head-tail joining protein *Vibrio* [WP_016786049.1]	100	4,00E-38
CDS_67	40445-42418	Terminase large subunit	Phage terminase large subunit family protein *Vibrio cortegadensis* [WP_261890222.1]	99	0.0
CDS_68	42306-42893	Terminase small subunit	Hypothetical protein *Vibrio cortegadensis* [WP_261890221.1]	100	4,00E-139

Table S3. Differences in biochemical profile of PF4 strain and lysogenic strain P1.1 of *V. anguillarum* with BIOLOG GEN III.

**BIOLOG Test**	***V. anguillarum*[ab]**		
	**PF4**	**P1.1**	
Gelatin	[light purple]	[white]	[green]
Tween 40	[light purple]	[white]	[green]
Glycyl-L-Proline	[light purple]	[purple]	[green]
D-Fructose	[light purple]	[purple]	[green]
α-Hydroxy-Butyric Acid	[white]	[light purple]	[green]
D-Trehalose	[purple]	[light purple]	[green]
β-Methyl-D Glucoside	[light purple]	[purple]	[green]
D-Gluconic Acid	[purple]	[white]	[green]
Acetic Acid	[purple]	[white]	[green]
Sodium Butyrate	[purple]	[white]	[red]
Potassium Tellurit	[purple]	[light purple]	[red]

[a] Purple color indicates a positive reaction (growth), white indicates negative reaction (no growth) and light purple indicates an intermediate reaction.

[b] The right column indicates whether the test is for susceptibility to the compound (red) or for its use as a carbon source (green).
